# Supplementary material for: Dynamics of Chromatin Opening across Larval Development in the Urochordate Ascidian Ciona savignyi
Source: Int J Mol Sci. 2024 Feb 28;25(5):2793. doi: 10.3390/ijms25052793 (PMC10931586; doi:10.3390/ijms25052793)
Supplement: Supplementary file 1 [file ijms-25-02793-s001.zip › ATAC-seq-hmc-s-2.27.pdf]

International Journal of *Molecular Sciences*

MDPI

Article

Dynamics of chromatin opening across larval development in the urochordate ascidian *Ciona savignyi*

Muchun He[1, 2, †], Yuting Li[1, †], Yajuan Li[1, 3], Bo Dong[1, 4, 5], Haiyan Yu[1, *]

1. Fang Zongxi Center for Marine EvoDevo, MoE Key Laboratory of Marine Genetics and Breeding, College of Marine Life Sciences, Ocean University of China, Qingdao 266003, China; hmc20160105@126.com (M.-C.H.); liyuting6876@stu.ouc.edu.cn (Y.-T.L.); lyj930722@126.com (Y.-J.L.); bodong@ouc.edu.cn (B.D.); haiyanyu@ouc.edu.cn (H.-Y. Y)
2. Liaoning Key Laboratory of Marine Animal Immunology, Dalian Ocean University, Dalian, 116023, China.
3. College of Animal Science, Inner Mongolia Agricultural University, Hohhot 010018, China.
4. Laboratory for Marine Biology and Biotechnology, Qingdao Marine Science and Technology Center, Qingdao 266237, China;
5. MoE Key Laboratory of Evolution & Marine Biodiversity, Institute of Evolution & Marine Biodiversity, Ocean University of China, Qingdao 266003, China.

† These authors contribute equally to this work.

* Correspondence: haiyanyu@ouc.edu.cn; Tel.: +86-0532-82032732

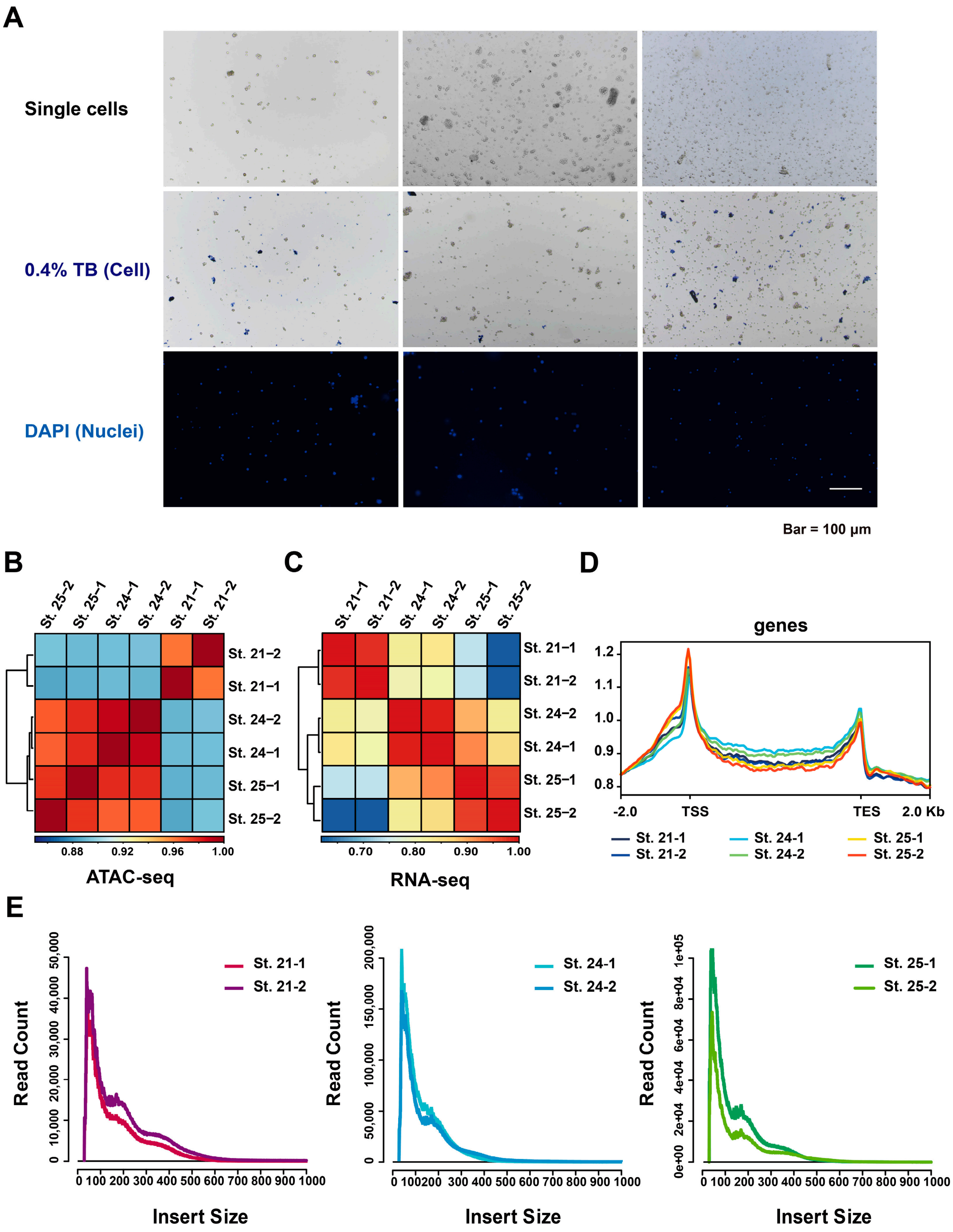

Figure S1: ATAC-seq quality control. (A) Sample preparation for ATAC-seq. The first row shows ascidian single cells, the second row shows the trypan blue staining results of single cells, and the third row shows the DAPI staining results of the nucleus. Scale bar represents 100 μm. (B) The Pearson's correlation results of ATAC-seq shown by a heat map. (C) The Pearson's correlation results of RNA-seq shown by a heat map. (D) Enrichment of ATAC-seq signals around the TSS and TES. The x-axis represents the normalized gene or peak length, and the y-axis represents the read enrichment. The larger the value, the higher the enrichment. TSS, transcription start site; TES, transcription end site. -2.0 represents 2 kb upstream of the TSS, and 2.0 represents 2 kb downstream of the TES. (E) Representative histogram of the frequency distribution of insert size from ATAC-sequencing.

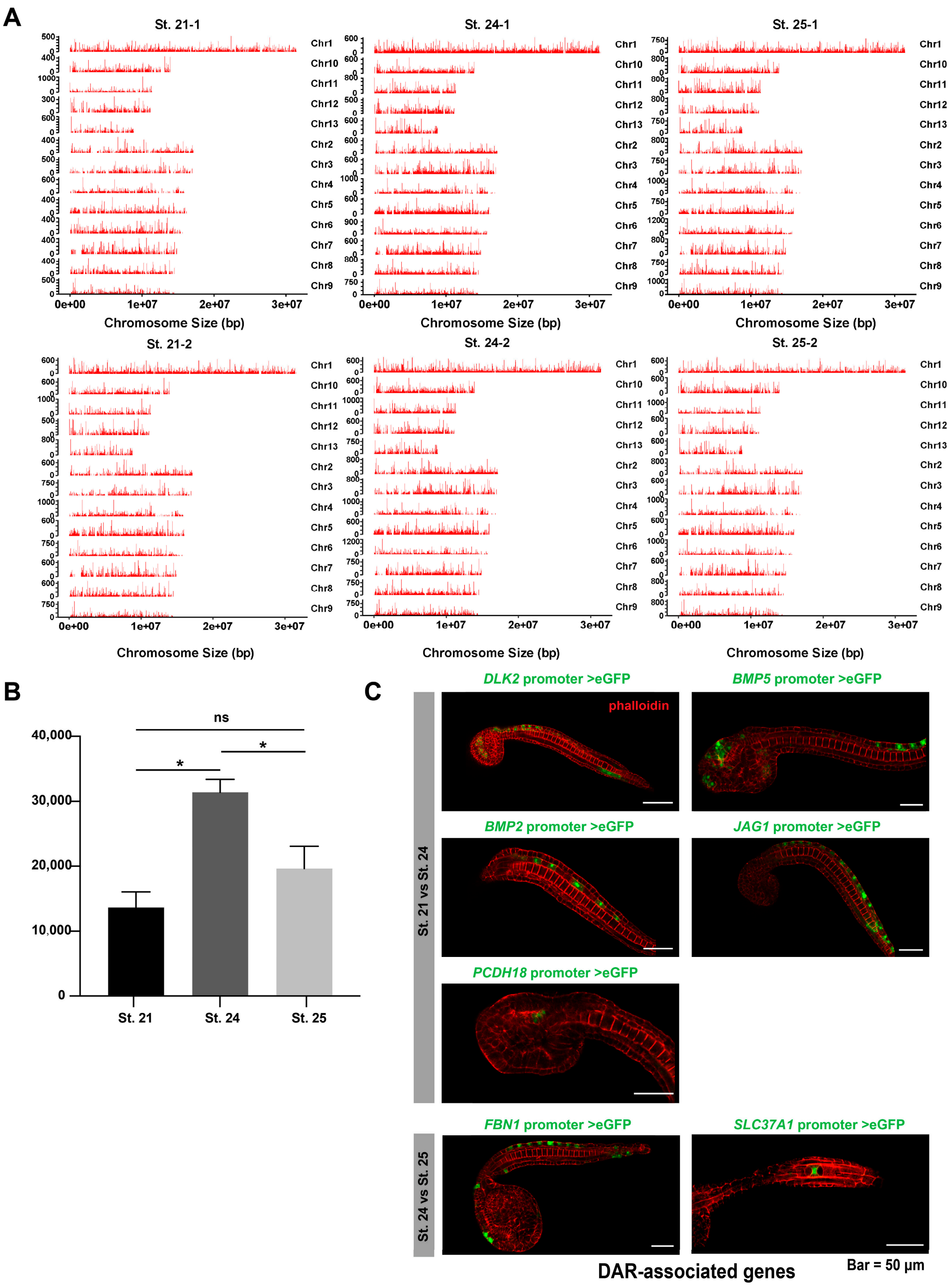

Figure S2: Analyses of the peaks. (A) Chromosomal distribution of all peak on the genomes. (B) The accessible peak numbers of each sample. Student's t-test was performed between each sample. Asterisks (∗) represent statistical significance ($P < 0.05$). (C) The tissue expression pattern of some DAR-associated genes at different stages by promoter assay.

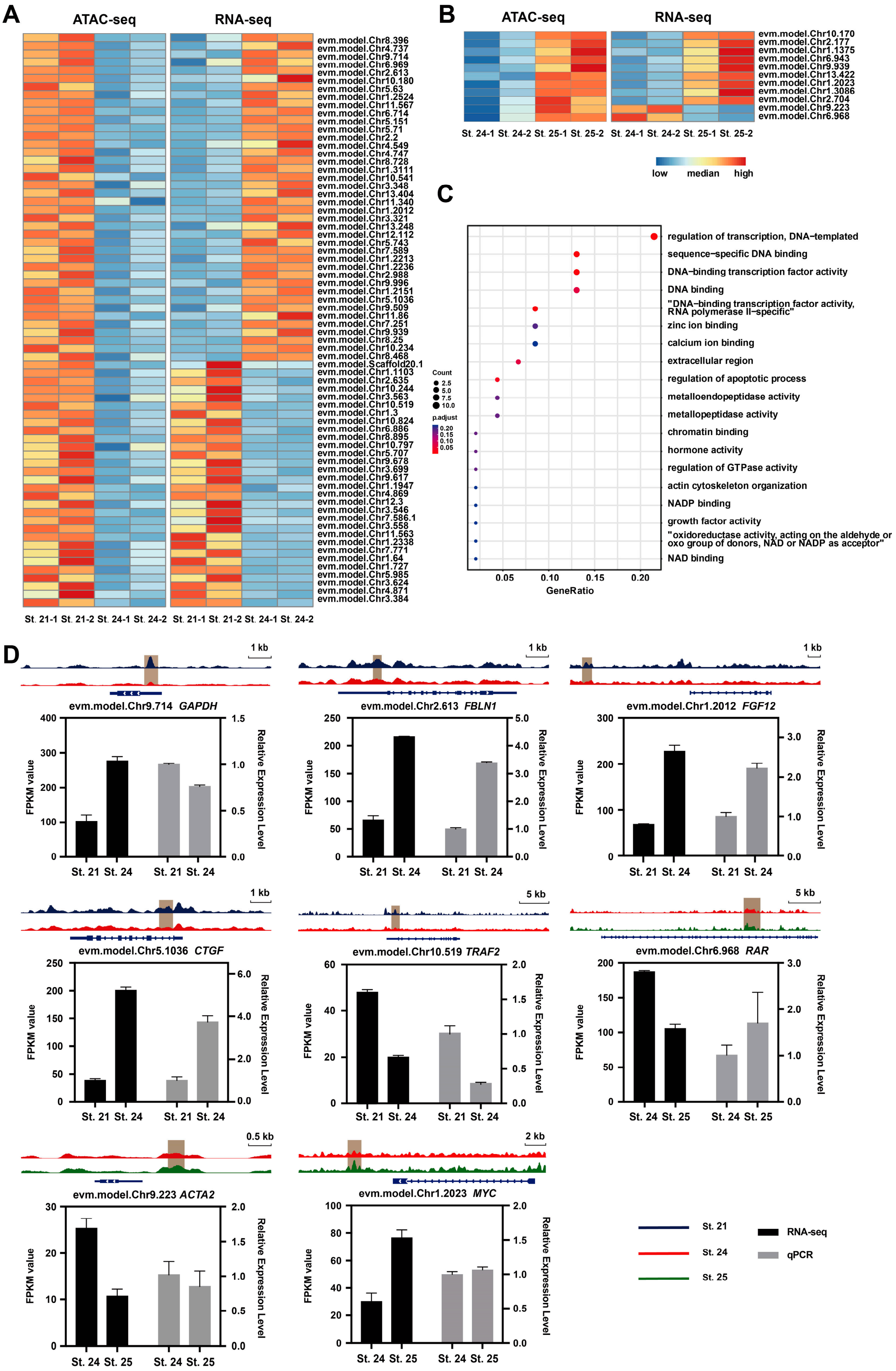

Figure S3: Integrated analysis of ATAC-seq and RNA-seq data. (A and B) Heat map showing the chromatin accessibility and expression level that were differentially expressed at different development stages. Each row means a gene. Genes were divided into two groups based on their chromatin accessibility (open to closed and closed to open). A: St. 21 vs. St. 24, B: St. 24 vs. St. 25. (C) GO enrichment analysis of the intersection genes at St. 21 vs. St. 24. (D) Relationship between accessibility and expression of genes.

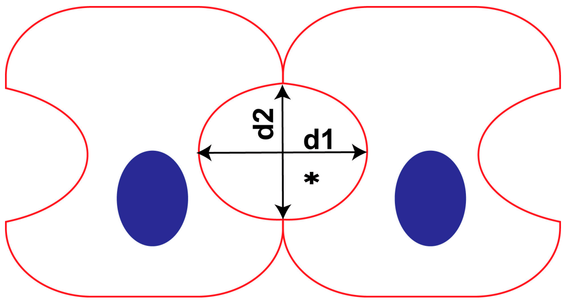

Figure S4: Measurement of notochord lumen volume of *Ciona*. The schematic images described spherical-lumen shape and the diameter of the lumen. d1 and d2 represent the diameter of antero-posterior (A-P) and dorsoventral (D-V), respectively.

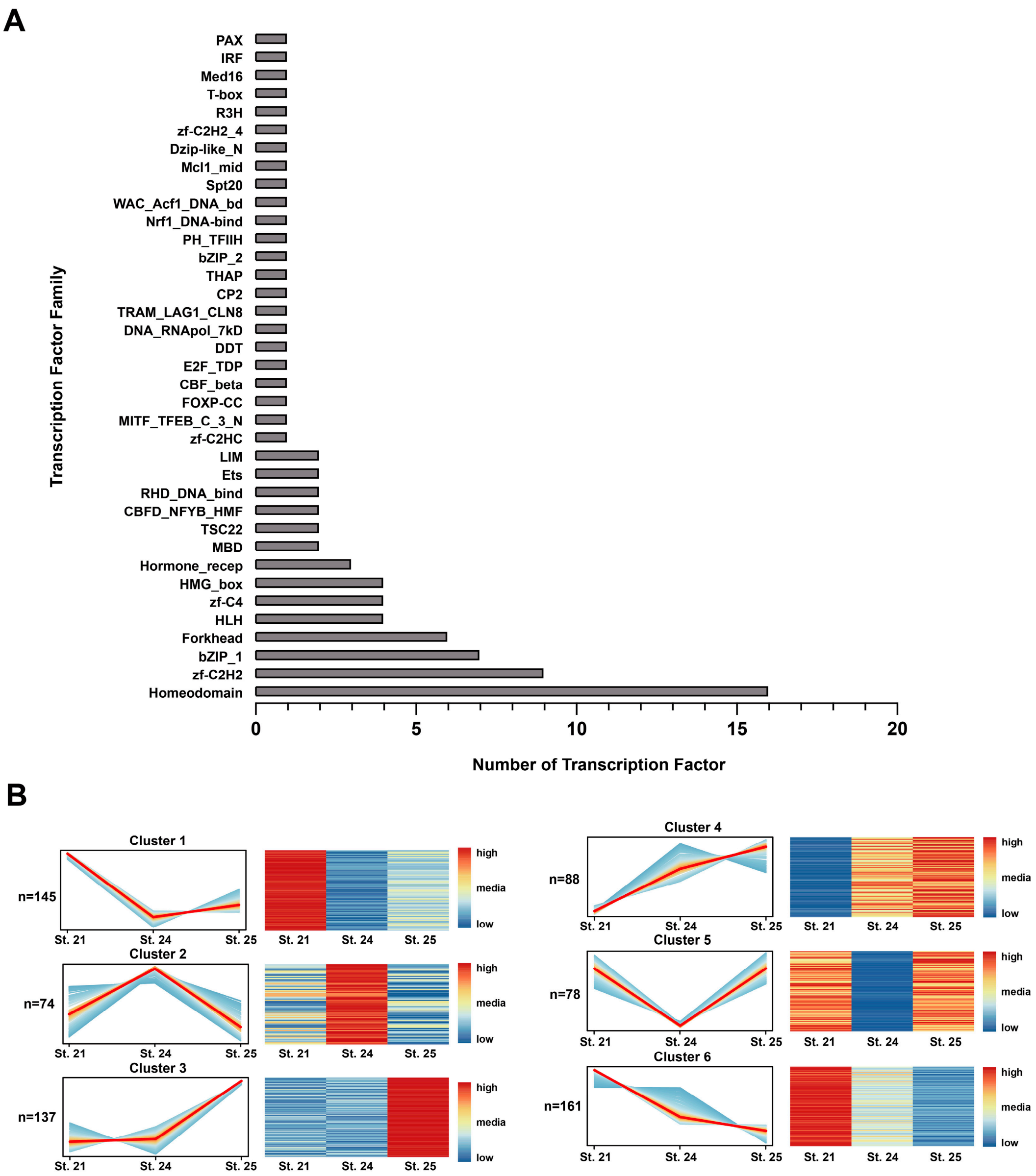

Figure S5: TFs and TF families in *C. savignyi*. (A) Statistics of transcription factors in transcription factor families in *C. savignyi* (Top 32). The horizontal coordinate represents the number of transcription factors, the vertical coordinate represents the names of transcription factor families. The number of transcription factors contained in each transcription factor family is indicated on the right side of the histogram. (B) Heat map of transcription factors expression cluster analysis. Each row represents one transcription factor. Expression change were grouped using K-means clustering. The numbers on the right indicate relative signal intensity.
